# {5-Methyl-1-[8-(trifluoro­meth­yl)quinolin-4-yl]-1*H*-1,2,3-triazol-4-yl}(morpholino)methanone

**DOI:** 10.1107/S1600536808037562

**Published:** 2008-11-20

**Authors:** N. Anuradha, A. Thiruvalluvar, M. Mahalinga, R. J. Butcher

**Affiliations:** a PG Research Department of Physics, Rajah Serfoji Government College (Autonomous), Thanjavur 613 005, Tamil Nadu, India; b SeQuent Scientific Limited, 120 A&B Industrial area, Baikampady, New Mangalore 575 011, India; c Department of Chemistry, Howard University, 525 College Street NW, Washington, DC 20059, USA

## Abstract

In the title mol­ecule, C_18_H_16_F_3_N_5_O_2_, the dihedral angle between the pyridine ring and the fused benzene ring is 4.50 (10)°. The triazole ring makes dihedral angles of 54.48 (12) and 57.91 (11)° with the pyridine and benzene rings, respectively. The morpholine ring atoms are disordered over two positions; the site-occupancy factors are *ca* 0.53 and 0.47. Inter­molecular C—H⋯F hydrogen bonding is found in the crystal structure. Furthermore, C—H⋯O and C—H⋯N intra­molecular contacts are also present.

## Related literature

For the uses of 1,2,3-triazoles and their benzo derivatives, see: Sanghvi *et al.* (1990 ▶). For a related crystal structure, see: Thiruvalluvar *et al.* (2007 ▶).
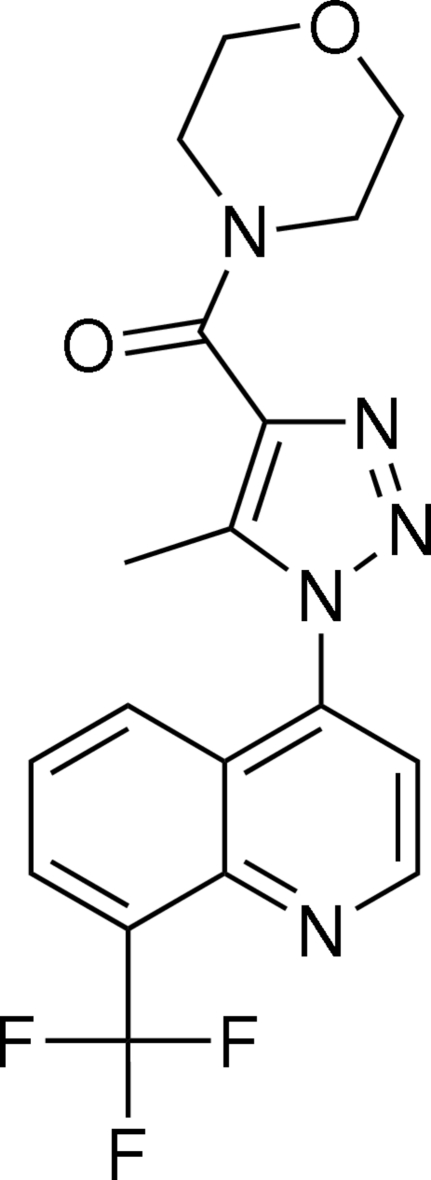


## Experimental

### 

#### Crystal data

C_18_H_16_F_3_N_5_O_2_
*M* *_r_* = 391.36
Triclinic, 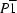

*a* = 9.2836 (15) Å
*b* = 9.6164 (11) Å
*c* = 9.9272 (11) Å
α = 92.082 (9)°
β = 93.063 (11)°
γ = 105.728 (12)°
*V* = 850.7 (2) Å^3^
*Z* = 2
Mo *K*α radiation
μ = 0.13 mm^−1^
*T* = 200 (2) K
0.53 × 0.28 × 0.22 mm

#### Data collection

Oxford Diffraction Gemini diffractometer
Absorption correction: multi-scan (*CrysAlis RED*; Oxford Diffraction, 2008 ▶) *T*_min_ = 0.934, *T*_max_ = 0.973
10917 measured reflections
5503 independent reflections
2919 reflections with *I* > 2σ(*I*)
*R*_int_ = 0.051

#### Refinement

*R*[*F* ^2^ > 2σ(*F* ^2^)] = 0.077
*wR*(*F* ^2^) = 0.262
*S* = 1.02
5503 reflections
300 parameters
H-atom parameters constrained
Δρ_max_ = 0.89 e Å^−3^
Δρ_min_ = −0.48 e Å^−3^

### 

Data collection: *CrysAlis CCD* (Oxford Diffraction, 2008 ▶); cell refinement: *CrysAlis RED* (Oxford Diffraction, 2008 ▶); data reduction: *CrysAlis RED*; program(s) used to solve structure: *SHELXS97* (Sheldrick, 2008 ▶); program(s) used to refine structure: *SHELXL97* (Sheldrick, 2008 ▶); molecular graphics: *ORTEP-3* (Farrugia, 1997 ▶); software used to prepare material for publication: *PLATON* (Spek, 2003 ▶).

## Supplementary Material

Crystal structure: contains datablocks global, I. DOI: 10.1107/S1600536808037562/bq2106sup1.cif

Structure factors: contains datablocks I. DOI: 10.1107/S1600536808037562/bq2106Isup2.hkl

Additional supplementary materials:  crystallographic information; 3D view; checkCIF report

## Figures and Tables

**Table 1:** Hydrogen-bond geometry (Å, °)

*D*—H⋯*A*	*D*—H	H⋯*A*	*D*⋯*A*	*D*—H⋯*A*
C3—H3⋯F1^i^	0.95	2.35	3.237 (3)	156
C16—H16*B*⋯O1	0.98	2.44	3.012 (3)	117
C23*A*—H23*B*⋯O1	0.99	2.36	2.812 (5)	107
C25*A*—H25*A*⋯N13	0.99	2.25	2.893 (5)	122

Symmetry code: (i) 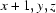.

**Fig. 1.**
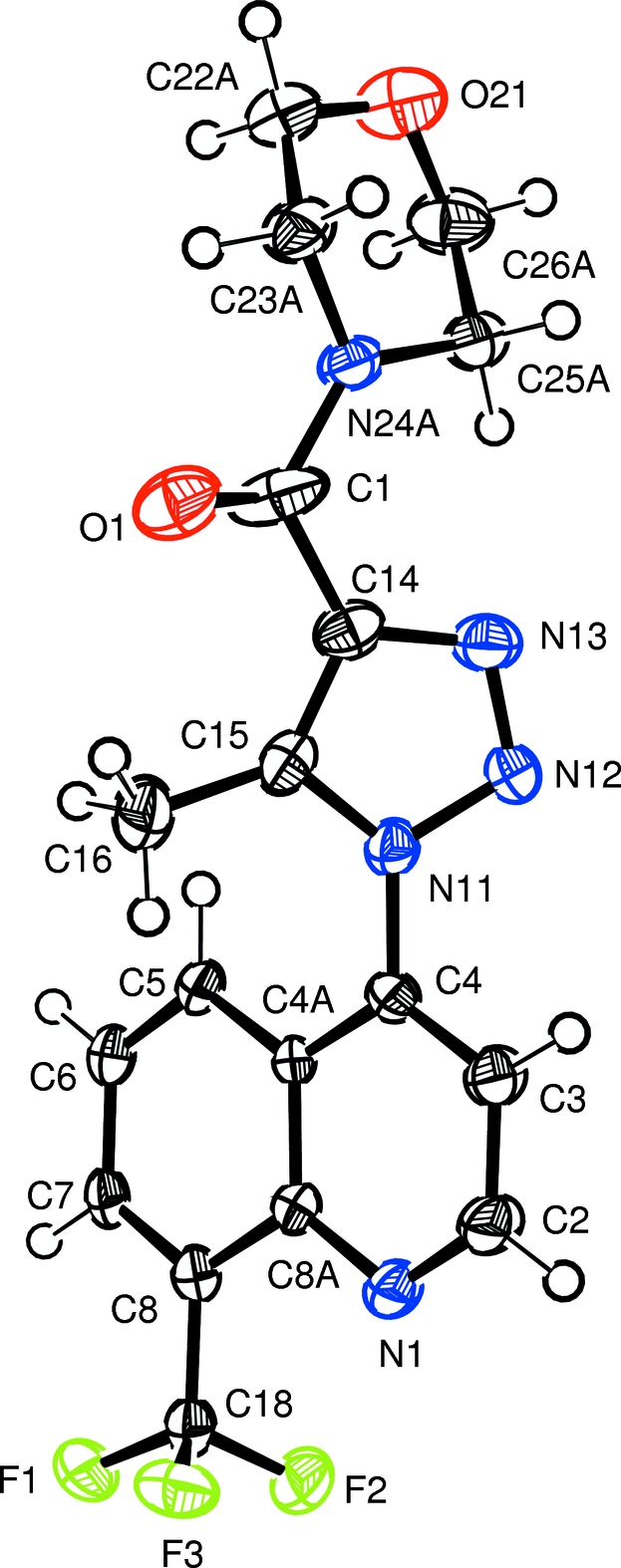
The molecular structure of the title compound, showing the atom-numbering scheme and displacement ellipsoids drawn at the 50% probability level. H atoms are shown as small spheres of arbitrary radius. Only the major component is shown.

**Fig. 2.**
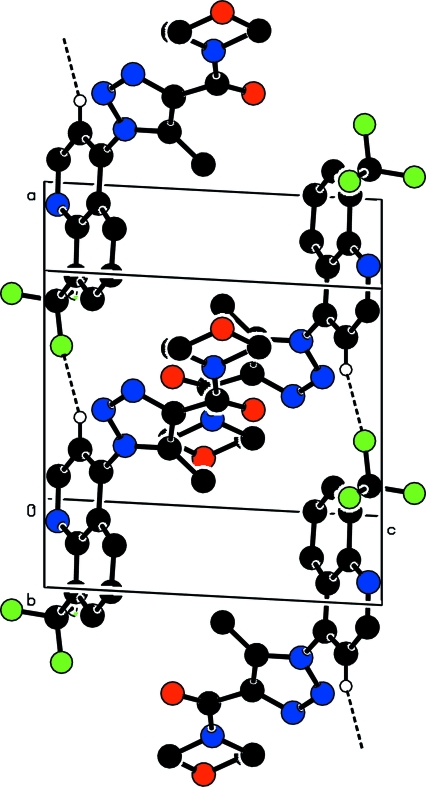
The packing of the title compound, viewed down the b axis. Dashed lines indicate hydrogen bonds. H atoms not involved in hydrogen bonding have been omitted. Only the major component is shown.

## supplementary crystallographic information

### Comment

1,2,3-Triazoles and their benzo derivatives have attracted considerable attention because of their theoretical interest and synthetic value. They also find numerous applications in industry and agriculture due to their extensive biological activities and successful application as fluorescent whiteners, light stabilizers and optical brightening agents (Sanghvi *et al.*, 1990). Thiruvalluvar *et al.* (2007) have reported the crystal structure of 1-{5-Methyl-1-[8-(trifluoromethyl)-quinolin-4-yl]-1*H*-1,2,3-triazol-4-yl}- ethanone.

In the title molecule, C_18_H_16_F_3_N_5_O_2_, Fig.1., the quinoline unit is nearly planar. The dihedral angle between the pyridine ring and the fused benzene ring is 4.50 (10)°. The triazole ring makes a dihedral angle of 54.48 (12)° and 57.91 (11)°, with that of pyridine and benzene rings, respectively. The trifluoromethyl group is coplanar with the attached benzene ring, except the fluoro atoms. The disordered morpholine ring is in chair form. Intermolecular C3—H3···F1(1 + *x*, *y*, *z*) hydrogen bond is found in the crystal structure. Further, C7—H7···F1, C16—H16B···O1, C23A—H23B···O1 and C25A—H25A···N13 intramolecular contacts are also found(Fig.2., Table 1).

### Experimental

5-Methyl-1-[8-(trifluoromethyl)quinolin-4-yl]-1*H*-1,2,3-triazole-4-carboxylic acid(10 g, 0.031 mol) was treated with thionyl chloride (3.7 g, 0.031 mol) in chlorofor (75 ml) and the mixture was heated to reflux for 2 h. The solvent was distilled out completely under vacuum. The residue was diluted with dry tetra hydrofuran (50 ml) and cooled to 273 K. This mixture was added slowly to the previously cooled morpholine in dry tetra hydrofuran (50 ml). Stirred for 1 h at 298 K and quenched to ice cooled water (500 ml). The precipitated solids were filtered, washed with water. The crude product was recrystallized from methanol. Yield 7.5 g (61.9%).

### Refinement

H atoms were positioned geometrically and allowed to ride on their parent atoms, with C—H = 0.95 - 0.99 Å and *U*_iso_(H) = 1.2 - 1.5 times *U*_eq_(C). The morpholine ring atoms are disordered over two positions; the site-occupancy factors refined to 0.534 (5) and 0.466 (5).

### Crystal data

**Table:**

C_18_H_16_F_3_N_5_O_2_	*Z* = 2
*M**_r_* = 391.36	*F*_000_ = 404
Triclinic, *P*1	*D*_x_ = 1.528 Mg m^−^^3^
Hall symbol: -P 1	Melting point: 464.5 K
*a* = 9.2836 (15) Å	Mo *K*α radiation λ = 0.71073 Å
*b* = 9.6164 (11) Å	Cell parameters from 4281 reflections
*c* = 9.9272 (11) Å	θ = 4.8–32.7º
α = 92.082 (9)º	µ = 0.13 mm^−^^1^
β = 93.063 (11)º	*T* = 200 (2) K
γ = 105.728 (12)º	Needle, colourless
*V* = 850.7 (2) Å^3^	0.53 × 0.28 × 0.22 mm

### Data collection

**Table:**

Oxford Diffraction Gemini diffractometer	5503 independent reflections
Radiation source: fine-focus sealed tube	2919 reflections with *I* > 2σ(*I*)
Monochromator: graphite	*R*_int_ = 0.051
Detector resolution: 10.5081 pixels mm^-1^	θ_max_ = 32.6º
*T* = 200(2) K	θ_min_ = 4.8º
φ and ω scans	*h* = −12→13
Absorption correction: multi-scan(CrysAlis RED; Oxford Diffraction, 2008)	*k* = −14→13
*T*_min_ = 0.934, *T*_max_ = 0.973	*l* = −14→14
10917 measured reflections	

### Refinement

**Table:**

Refinement on *F*^2^	Secondary atom site location: difference Fourier map
Least-squares matrix: full	Hydrogen site location: inferred from neighbouring sites
*R*[*F*^2^ > 2σ(*F*^2^)] = 0.077	H-atom parameters constrained
*wR*(*F*^2^) = 0.262	*w* = 1/[σ^2^(*F*_o_^2^) + (0.1618*P*)^2^] where *P* = (*F*_o_^2^ + 2*F*_c_^2^)/3
*S* = 1.02	(Δ/σ)_max_ = 0.001
5503 reflections	Δρ_max_ = 0.89 e Å^−^^3^
300 parameters	Δρ_min_ = −0.48 e Å^−^^3^
Primary atom site location: structure-invariant direct methods	Extinction correction: none

### Special details

**Table:**

Geometry. Bond distances, angles *etc*. have been calculated using the rounded fractional coordinates. All su's are estimated from the variances of the (full) variance-covariance matrix. The cell e.s.d.'s are taken into account in the estimation of distances, angles and torsion angles
Refinement. Refinement of *F*^2^ against ALL reflections. The weighted *R*-factor *wR* and goodness of fit *S* are based on *F*^2^, conventional *R*-factors *R* are based on *F*, with *F* set to zero for negative *F*^2^. The threshold expression of *F*^2^ > 2σ(*F*^2^) is used only for calculating *R*-factors(gt) *etc*. and is not relevant to the choice of reflections for refinement. *R*-factors based on *F*^2^ are statistically about twice as large as those based on *F*, and *R*- factors based on ALL data will be even larger.

### Fractional atomic coordinates and isotropic or equivalent isotropic displacement parameters (Å^2^)

**Table:**

	*x*	*y*	*z*	*U*_iso_*/*U*_eq_	Occ. (<1)
F1	−0.46264 (15)	0.18931 (16)	0.05088 (17)	0.0495 (5)	
F2	−0.31631 (17)	0.15031 (16)	−0.09678 (13)	0.0450 (5)	
F3	−0.31915 (17)	0.05319 (14)	0.09537 (15)	0.0463 (5)	
O1	0.5096 (2)	0.69496 (19)	0.61981 (17)	0.0461 (6)	
O21	0.8837 (3)	1.1342 (2)	0.5276 (2)	0.0574 (7)	
N1	−0.0181 (2)	0.18471 (18)	0.03929 (19)	0.0309 (5)	
N11	0.3262 (2)	0.52201 (19)	0.23910 (18)	0.0281 (5)	
N12	0.4534 (2)	0.5836 (2)	0.17560 (19)	0.0328 (5)	
N13	0.5437 (2)	0.6728 (2)	0.2630 (2)	0.0359 (6)	
N24A	0.6853 (5)	0.8662 (4)	0.5018 (4)	0.0310 (10)	0.534 (5)
C1	0.5494 (3)	0.7483 (2)	0.5121 (3)	0.0458 (8)	
C2	0.1246 (3)	0.1857 (2)	0.0423 (3)	0.0377 (7)	
C3	0.2428 (3)	0.2962 (2)	0.1060 (3)	0.0353 (7)	
C4	0.2085 (2)	0.4121 (2)	0.1684 (2)	0.0254 (5)	
C4A	0.0590 (2)	0.42273 (19)	0.16018 (18)	0.0215 (5)	
C5	0.0162 (2)	0.5470 (2)	0.2076 (2)	0.0267 (6)	
C6	−0.1308 (3)	0.5473 (2)	0.1979 (2)	0.0306 (6)	
C7	−0.2428 (3)	0.4259 (2)	0.1427 (2)	0.0302 (6)	
C8	−0.2055 (2)	0.3047 (2)	0.0935 (2)	0.0271 (6)	
C8A	−0.0522 (2)	0.3025 (2)	0.09725 (19)	0.0236 (5)	
C14	0.4783 (3)	0.6685 (2)	0.3837 (2)	0.0320 (6)	
C15	0.3391 (3)	0.5699 (2)	0.3709 (2)	0.0303 (6)	
C16	0.2255 (3)	0.5135 (3)	0.4714 (2)	0.0422 (8)	
C18	−0.3250 (3)	0.1757 (2)	0.0361 (2)	0.0326 (6)	
C22A	0.8185 (12)	1.0861 (9)	0.6366 (10)	0.047 (2)	0.534 (5)
C23A	0.7731 (6)	0.9247 (5)	0.6284 (4)	0.0395 (16)	0.534 (5)
C25A	0.7564 (6)	0.9318 (5)	0.3820 (4)	0.0370 (14)	0.534 (5)
C26A	0.8030 (12)	1.0939 (9)	0.4013 (10)	0.046 (2)	0.534 (5)
C23B	0.6638 (6)	0.9736 (6)	0.6483 (5)	0.0366 (16)	0.466 (5)
N24B	0.6066 (6)	0.8915 (4)	0.5195 (4)	0.0328 (14)	0.466 (5)
C22B	0.8321 (12)	1.0331 (9)	0.6461 (12)	0.046 (3)	0.466 (5)
C25B	0.6442 (6)	0.9771 (5)	0.4021 (5)	0.0327 (14)	0.466 (5)
C26B	0.8137 (12)	1.0426 (11)	0.4118 (12)	0.049 (3)	0.466 (5)
H2	0.14907	0.10638	−0.00156	0.0452*	
H16B	0.27710	0.50786	0.55891	0.0633*	
H16C	0.16359	0.41696	0.43998	0.0633*	
H22A	0.72904	1.12206	0.64610	0.0556*	0.534 (5)
H22B	0.88847	1.12224	0.71694	0.0556*	0.534 (5)
H23A	0.86388	0.88951	0.63496	0.0473*	0.534 (5)
H23B	0.71271	0.88908	0.70565	0.0473*	0.534 (5)
H25A	0.68507	0.90210	0.30172	0.0446*	0.534 (5)
H25B	0.84542	0.89691	0.36623	0.0446*	0.534 (5)
H26A	0.86668	1.13632	0.32811	0.0556*	0.534 (5)
H26B	0.71325	1.13077	0.39766	0.0556*	0.534 (5)
H3	0.34351	0.29084	0.10601	0.0423*	
H5	0.09032	0.62952	0.24602	0.0320*	
H6	−0.15791	0.63074	0.22882	0.0368*	
H7	−0.34488	0.42725	0.13926	0.0362*	
H16A	0.16161	0.57888	0.48100	0.0633*	
H22C	0.87722	0.95111	0.63922	0.0551*	0.466 (5)
H22D	0.87115	1.08795	0.73275	0.0551*	0.466 (5)
H23C	0.63897	0.90964	0.72455	0.0439*	0.466 (5)
H23D	0.61694	1.05388	0.66020	0.0439*	0.466 (5)
H25C	0.59250	1.05464	0.40105	0.0389*	0.466 (5)
H25D	0.61240	0.91513	0.31811	0.0389*	0.466 (5)
H26C	0.86056	0.96167	0.40598	0.0586*	0.466 (5)
H26D	0.83967	1.09899	0.33083	0.0586*	0.466 (5)

### Atomic displacement parameters (Å^2^)

**Table:**

	*U*^11^	*U*^22^	*U*^33^	*U*^12^	*U*^13^	*U*^23^
F1	0.0213 (7)	0.0542 (9)	0.0675 (11)	0.0026 (6)	0.0085 (7)	−0.0172 (8)
F2	0.0394 (8)	0.0581 (9)	0.0325 (7)	0.0072 (7)	0.0008 (6)	−0.0138 (6)
F3	0.0431 (9)	0.0326 (7)	0.0543 (9)	−0.0039 (6)	−0.0011 (7)	0.0018 (6)
O1	0.0593 (12)	0.0418 (9)	0.0343 (9)	0.0112 (8)	−0.0081 (8)	−0.0002 (7)
O21	0.0619 (14)	0.0487 (11)	0.0546 (12)	0.0067 (10)	−0.0078 (11)	−0.0056 (9)
N1	0.0293 (10)	0.0270 (8)	0.0373 (10)	0.0100 (7)	0.0030 (8)	−0.0062 (7)
N11	0.0242 (9)	0.0324 (8)	0.0283 (8)	0.0096 (7)	0.0017 (7)	−0.0047 (7)
N12	0.0233 (9)	0.0377 (9)	0.0363 (10)	0.0067 (7)	0.0048 (8)	−0.0052 (8)
N13	0.0306 (10)	0.0340 (9)	0.0394 (11)	0.0045 (8)	−0.0031 (8)	−0.0040 (8)
N24A	0.035 (2)	0.0281 (17)	0.0241 (17)	−0.0003 (15)	0.0002 (16)	−0.0037 (13)
C1	0.0709 (19)	0.0238 (10)	0.0342 (12)	0.0035 (11)	−0.0191 (12)	−0.0020 (9)
C2	0.0310 (12)	0.0310 (10)	0.0527 (14)	0.0135 (9)	0.0012 (10)	−0.0125 (9)
C3	0.0242 (10)	0.0368 (11)	0.0465 (13)	0.0134 (9)	−0.0006 (10)	−0.0109 (9)
C4	0.0191 (9)	0.0281 (9)	0.0280 (10)	0.0058 (7)	0.0001 (8)	−0.0031 (7)
C4A	0.0184 (9)	0.0226 (8)	0.0224 (9)	0.0037 (7)	0.0057 (7)	−0.0029 (7)
C5	0.0263 (10)	0.0282 (9)	0.0258 (10)	0.0084 (8)	0.0041 (8)	−0.0063 (7)
C6	0.0296 (11)	0.0333 (10)	0.0320 (11)	0.0132 (9)	0.0081 (9)	−0.0029 (8)
C7	0.0223 (10)	0.0386 (11)	0.0309 (11)	0.0096 (8)	0.0093 (8)	−0.0038 (8)
C8	0.0240 (10)	0.0307 (10)	0.0262 (10)	0.0063 (8)	0.0063 (8)	−0.0003 (7)
C8A	0.0243 (10)	0.0230 (8)	0.0237 (9)	0.0068 (7)	0.0039 (8)	−0.0019 (7)
C14	0.0374 (12)	0.0269 (10)	0.0320 (11)	0.0114 (9)	−0.0052 (9)	−0.0015 (8)
C15	0.0350 (12)	0.0339 (10)	0.0250 (10)	0.0153 (9)	0.0003 (9)	−0.0033 (8)
C16	0.0407 (14)	0.0551 (14)	0.0297 (11)	0.0106 (11)	0.0080 (10)	−0.0019 (10)
C18	0.0264 (11)	0.0381 (11)	0.0326 (11)	0.0079 (9)	0.0068 (9)	−0.0061 (9)
C22A	0.047 (4)	0.043 (4)	0.044 (3)	0.005 (3)	−0.001 (3)	−0.013 (4)
C23A	0.043 (3)	0.046 (3)	0.024 (2)	0.004 (2)	−0.0029 (19)	−0.0016 (17)
C25A	0.038 (3)	0.036 (2)	0.031 (2)	0.0001 (19)	0.0040 (19)	−0.0035 (17)
C26A	0.049 (4)	0.044 (4)	0.041 (3)	0.006 (3)	−0.010 (3)	0.006 (3)
C23B	0.029 (3)	0.047 (3)	0.030 (2)	0.005 (2)	0.004 (2)	−0.008 (2)
N24B	0.037 (3)	0.031 (2)	0.026 (2)	0.0027 (19)	0.0019 (19)	−0.0058 (15)
C22B	0.041 (4)	0.040 (5)	0.049 (4)	0.002 (4)	−0.012 (3)	−0.007 (4)
C25B	0.030 (2)	0.029 (2)	0.036 (3)	0.0027 (18)	0.001 (2)	0.0031 (18)
C26B	0.042 (4)	0.059 (7)	0.047 (4)	0.010 (4)	0.017 (3)	0.018 (5)

### Geometric parameters (Å, °)

**Table:**

F1—C18	1.334 (3)	C8—C8A	1.428 (3)
F2—C18	1.344 (2)	C14—C15	1.376 (3)
F3—C18	1.349 (2)	C15—C16	1.498 (3)
O1—C1	1.234 (3)	C22A—C23A	1.492 (10)
O21—C22A	1.306 (10)	C22B—C23B	1.513 (12)
O21—C26A	1.411 (10)	C25A—C26A	1.503 (10)
O21—C22B	1.565 (11)	C25B—C26B	1.525 (13)
O21—C26B	1.434 (12)	C2—H2	0.9500
N1—C2	1.321 (3)	C3—H3	0.9500
N1—C8A	1.371 (3)	C5—H5	0.9500
N11—N12	1.368 (3)	C6—H6	0.9500
N11—C4	1.429 (3)	C7—H7	0.9500
N11—C15	1.359 (3)	C16—H16A	0.9800
N12—N13	1.292 (3)	C16—H16B	0.9800
N13—C14	1.369 (3)	C16—H16C	0.9800
N24A—C1	1.461 (5)	C22A—H22A	0.9900
N24A—C23A	1.467 (6)	C22A—H22B	0.9900
N24A—C25A	1.470 (6)	C22B—H22D	0.9900
N24B—C1	1.333 (4)	C22B—H22C	0.9900
N24B—C23B	1.472 (6)	C23A—H23B	0.9900
N24B—C25B	1.454 (6)	C23A—H23A	0.9900
C1—C14	1.488 (3)	C23B—H23D	0.9900
C2—C3	1.406 (3)	C23B—H23C	0.9900
C3—C4	1.375 (3)	C25A—H25B	0.9900
C4—C4A	1.417 (3)	C25A—H25A	0.9900
C4A—C5	1.430 (3)	C25B—H25D	0.9900
C4A—C8A	1.423 (3)	C25B—H25C	0.9900
C5—C6	1.364 (3)	C26A—H26B	0.9900
C6—C7	1.407 (3)	C26A—H26A	0.9900
C7—C8	1.383 (3)	C26B—H26C	0.9900
C8—C18	1.493 (3)	C26B—H26D	0.9900
			
F1···C3^i^	3.237 (3)	C4A···H16C	2.9000
F1···N12^ii^	3.179 (3)	C5···H16A	2.9300
F1···F3^iii^	2.920 (2)	C5···H16C	3.0900
F2···C22B^iv^	3.270 (12)	C6···H16B^xi^	2.8300
F2···C23B^iv^	2.965 (5)	C7···H16B^xi^	3.0600
F2···N1	2.945 (3)	C8···H26A^v^	3.0300
F2···C22A^iv^	3.100 (10)	C8A···H26A^v^	2.8600
F3···C26A^v^	3.157 (10)	C14···H23D^viii^	3.0700
F3···F1^iii^	2.920 (2)	C14···H25A	2.7100
F3···C25B^v^	3.172 (5)	C14···H25D	2.4900
F3···N1	2.832 (2)	C15···H5	2.7700
F3···C25A^v^	3.229 (4)	C16···H5	2.9100
F3···C26B^v^	3.328 (12)	C22A···H5^viii^	2.8200
F1···H7	2.3700	C22A···H25B^ix^	3.0800
F1···H3^i^	2.3500	C22B···H26C^ix^	2.9100
F2···H22D^iv^	2.6600	C23B···H25D^viii^	3.0600
F2···H22A^iv^	2.6300	C23B···H25C^viii^	2.3400
F2···H23D^iv^	2.5400	C25B···H23D^viii^	2.4000
F2···H23C^iv^	2.7900	C25B···H25C^viii^	2.9800
F2···H22B^iv^	2.7800	C26A···H23A^ix^	3.0900
F3···H26A^v^	2.7700	C26B···H22C^ix^	2.9300
F3···H25D^v^	2.6400	H2···F3^vi^	2.6600
F3···H26D^v^	2.6500	H2···N1^vi^	2.7400
F3···H25A^v^	2.5600	H3···H23B^vii^	2.5700
F3···H2^vi^	2.6600	H3···F1^x^	2.3500
O1···C16	3.012 (3)	H3···N12	2.7700
O1···N11^vii^	3.217 (3)	H5···N11	2.6700
O1···C15^vii^	3.227 (3)	H5···H16A	2.4900
O21···N24B	2.963 (5)	H5···C15	2.7700
O21···N24A	2.726 (4)	H5···C16	2.9100
O1···H23C	2.2700	H5···C22A^viii^	2.8200
O1···H23B	2.3600	H5···H22B^viii^	2.3600
O1···H25C^viii^	2.8300	H6···N13^i^	2.9400
O1···H16B	2.4400	H7···F1	2.3700
O21···H16A^viii^	2.9100	H7···N12^i^	2.7300
O21···H25B^ix^	2.7700	H16A···C5	2.9300
O21···H26C^ix^	2.8300	H16A···H5	2.4900
N1···F2	2.945 (3)	H16A···O21^viii^	2.9100
N1···F3	2.832 (2)	H16B···C6^xi^	2.8300
N11···O1^vii^	3.217 (3)	H16B···C7^xi^	3.0600
N12···F1^ii^	3.179 (3)	H16B···C1	3.0000
N13···C25A	2.893 (5)	H16B···O1	2.4400
N13···C25B	3.071 (5)	H16C···C4A	2.9000
N13···N24B	3.168 (4)	H16C···C5	3.0900
N13···N24A	2.971 (4)	H16C···C4	2.7500
N24A···N13	2.971 (4)	H22A···H26B	2.4700
N24A···O21	2.726 (4)	H22A···F2^xii^	2.6300
N24B···N24B^viii^	3.262 (7)	H22B···H5^viii^	2.3600
N24B···O21	2.963 (5)	H22B···F2^xii^	2.7800
N24B···N13	3.168 (4)	H22C···H26C	2.3200
N24B···C25B^viii^	3.056 (8)	H22C···C26B^ix^	2.9300
N1···H2^vi^	2.7400	H22C···H26C^ix^	2.4200
N11···H5	2.6700	H22D···F2^xii^	2.6600
N12···H7^x^	2.7300	H23A···H26A^ix^	2.5900
N12···H3	2.7700	H23A···C26A^ix^	3.0900
N13···H25A	2.2500	H23B···O1	2.3600
N13···H6^x^	2.9400	H23B···C2^vii^	3.0600
N13···H25D	2.2800	H23B···H3^vii^	2.5700
N24B···H23D^viii^	2.8300	H23B···C3^vii^	2.7200
N24B···H25C^viii^	2.2300	H23C···O1	2.2700
C2···C6^ii^	3.558 (3)	H23C···F2^xii^	2.7900
C3···F1^x^	3.237 (3)	H23C···C3^vii^	3.0300
C3···C23A^vii^	3.430 (5)	H23C···H25C^viii^	2.5400
C4···C7^ii^	3.500 (3)	H23D···H25C	2.5700
C4A···C16	3.355 (3)	H23D···F2^xii^	2.5400
C5···C8A^ii^	3.395 (3)	H23D···H25D^viii^	2.2500
C5···C15	3.280 (3)	H23D···N24B^viii^	2.8300
C5···C16	3.255 (3)	H23D···C25B^viii^	2.4000
C6···C16^xi^	3.473 (3)	H23D···C14^viii^	3.0700
C6···C2^ii^	3.558 (3)	H23D···H25C^viii^	1.9900
C7···C4^ii^	3.500 (3)	H25A···N13	2.2500
C8A···C5^ii^	3.395 (3)	H25A···C14	2.7100
C15···C5	3.280 (3)	H25A···F3^xiii^	2.5600
C15···O1^vii^	3.227 (3)	H25B···C22A^ix^	3.0800
C16···C5	3.255 (3)	H25B···O21^ix^	2.7700
C16···C6^xi^	3.473 (3)	H25C···O1^viii^	2.8300
C16···O1	3.012 (3)	H25C···H23D	2.5700
C16···C4A	3.355 (3)	H25C···C25B^viii^	2.9800
C22A···F2^xii^	3.100 (10)	H25C···H23C^viii^	2.5400
C22B···F2^xii^	3.270 (12)	H25C···H23D^viii^	1.9900
C23A···C3^vii^	3.430 (5)	H25C···N24B^viii^	2.2300
C23B···F2^xii^	2.965 (5)	H25C···C23B^viii^	2.3400
C23B···C25B^viii^	3.041 (8)	H25C···C1^viii^	2.7300
C25A···F3^xiii^	3.229 (4)	H25D···F3^xiii^	2.6400
C25A···N13	2.893 (5)	H25D···N13	2.2800
C25B···N13	3.071 (5)	H25D···H23D^viii^	2.2500
C25B···F3^xiii^	3.172 (5)	H25D···C14	2.4900
C25B···C23B^viii^	3.041 (8)	H25D···C23B^viii^	3.0600
C25B···C25B^viii^	3.503 (8)	H26A···C8^xiii^	3.0300
C25B···N24B^viii^	3.056 (8)	H26A···F3^xiii^	2.7700
C26A···F3^xiii^	3.157 (10)	H26A···C8A^xiii^	2.8600
C26B···F3^xiii^	3.328 (12)	H26A···H23A^ix^	2.5900
C1···H25C^viii^	2.7300	H26B···H22A	2.4700
C1···H16B	3.0000	H26C···H22C	2.3200
C2···H23B^vii^	3.0600	H26C···O21^ix^	2.8300
C3···H23C^vii^	3.0300	H26C···C22B^ix^	2.9100
C3···H23B^vii^	2.7200	H26C···H22C^ix^	2.4200
C4···H16C	2.7500	H26D···F3^xiii^	2.6500
			
C22A—O21—C26A	118.7 (6)	N1—C2—H2	118.00
C22B—O21—C26B	101.9 (6)	C3—C2—H2	118.00
C2—N1—C8A	117.59 (18)	C2—C3—H3	121.00
N12—N11—C4	119.49 (17)	C4—C3—H3	121.00
N12—N11—C15	111.24 (18)	C4A—C5—H5	120.00
C4—N11—C15	129.00 (19)	C6—C5—H5	120.00
N11—N12—N13	106.99 (17)	C5—C6—H6	119.00
N12—N13—C14	109.29 (19)	C7—C6—H6	120.00
C1—N24A—C23A	116.8 (3)	C6—C7—H7	120.00
C1—N24A—C25A	130.2 (4)	C8—C7—H7	120.00
C23A—N24A—C25A	112.9 (4)	C15—C16—H16A	109.00
C1—N24B—C23B	122.3 (4)	C15—C16—H16B	109.00
C1—N24B—C25B	123.6 (4)	C15—C16—H16C	109.00
C23B—N24B—C25B	113.2 (4)	H16A—C16—H16B	109.00
N24A—C1—C14	116.7 (3)	H16A—C16—H16C	109.00
N24B—C1—C14	121.6 (3)	H16B—C16—H16C	110.00
O1—C1—C14	118.48 (19)	O21—C22A—H22A	110.00
O1—C1—N24B	114.9 (3)	O21—C22A—H22B	110.00
O1—C1—N24A	123.2 (3)	C23A—C22A—H22A	110.00
N1—C2—C3	124.4 (2)	C23A—C22A—H22B	110.00
C2—C3—C4	118.1 (2)	H22A—C22A—H22B	108.00
C3—C4—C4A	120.16 (19)	O21—C22B—H22C	109.00
N11—C4—C4A	120.93 (17)	O21—C22B—H22D	109.00
N11—C4—C3	118.90 (19)	C23B—C22B—H22C	109.00
C5—C4A—C8A	119.32 (17)	C23B—C22B—H22D	109.00
C4—C4A—C8A	116.74 (17)	H22C—C22B—H22D	108.00
C4—C4A—C5	123.92 (17)	H23A—C23A—H23B	108.00
C4A—C5—C6	120.15 (17)	N24A—C23A—H23B	109.00
C5—C6—C7	121.0 (2)	C22A—C23A—H23A	109.00
C6—C7—C8	120.5 (2)	N24A—C23A—H23A	109.00
C8A—C8—C18	119.66 (18)	C22A—C23A—H23B	109.00
C7—C8—C8A	120.05 (19)	N24B—C23B—H23D	110.00
C7—C8—C18	120.3 (2)	C22B—C23B—H23C	110.00
N1—C8A—C4A	122.68 (18)	N24B—C23B—H23C	110.00
N1—C8A—C8	118.56 (17)	H23C—C23B—H23D	108.00
C4A—C8A—C8	118.76 (17)	C22B—C23B—H23D	110.00
N13—C14—C1	126.0 (2)	N24A—C25A—H25B	110.00
C1—C14—C15	124.6 (2)	N24A—C25A—H25A	110.00
N13—C14—C15	109.24 (18)	H25A—C25A—H25B	108.00
C14—C15—C16	132.13 (19)	C26A—C25A—H25A	110.00
N11—C15—C16	124.6 (2)	C26A—C25A—H25B	110.00
N11—C15—C14	103.20 (19)	N24B—C25B—H25D	110.00
F1—C18—F2	106.17 (19)	H25C—C25B—H25D	109.00
F1—C18—F3	106.26 (18)	C26B—C25B—H25C	110.00
F1—C18—C8	112.44 (17)	C26B—C25B—H25D	110.00
F3—C18—C8	112.44 (19)	N24B—C25B—H25C	110.00
F2—C18—F3	106.18 (16)	O21—C26A—H26A	110.00
F2—C18—C8	112.84 (18)	O21—C26A—H26B	110.00
O21—C22A—C23A	109.8 (7)	C25A—C26A—H26A	110.00
O21—C22B—C23B	114.1 (7)	C25A—C26A—H26B	110.00
N24A—C23A—C22A	111.7 (5)	H26A—C26A—H26B	108.00
N24B—C23B—C22B	108.5 (6)	O21—C26B—H26C	107.00
N24A—C25A—C26A	110.5 (5)	O21—C26B—H26D	107.00
N24B—C25B—C26B	107.5 (6)	C25B—C26B—H26C	107.00
O21—C26A—C25A	109.2 (6)	C25B—C26B—H26D	108.00
O21—C26B—C25B	119.4 (8)	H26C—C26B—H26D	107.00
			
C26A—O21—C22A—C23A	58.9 (10)	C2—C3—C4—C4A	−4.0 (3)
C22A—O21—C26A—C25A	−58.4 (10)	N11—C4—C4A—C5	6.7 (3)
C8A—N1—C2—C3	2.9 (4)	N11—C4—C4A—C8A	−175.01 (17)
C2—N1—C8A—C4A	−0.5 (3)	C3—C4—C4A—C5	−172.2 (2)
C2—N1—C8A—C8	179.1 (2)	C3—C4—C4A—C8A	6.1 (3)
C4—N11—N12—N13	−176.69 (18)	C4—C4A—C5—C6	−178.86 (19)
C15—N11—N12—N13	−2.2 (2)	C8A—C4A—C5—C6	2.9 (3)
N12—N11—C4—C3	51.3 (3)	C4—C4A—C8A—N1	−3.9 (3)
N12—N11—C4—C4A	−127.7 (2)	C4—C4A—C8A—C8	176.46 (17)
C15—N11—C4—C3	−122.1 (3)	C5—C4A—C8A—N1	174.44 (18)
C15—N11—C4—C4A	59.0 (3)	C5—C4A—C8A—C8	−5.2 (3)
N12—N11—C15—C14	2.4 (2)	C4A—C5—C6—C7	0.6 (3)
N12—N11—C15—C16	−174.1 (2)	C5—C6—C7—C8	−1.8 (3)
C4—N11—C15—C14	176.2 (2)	C6—C7—C8—C8A	−0.6 (3)
C4—N11—C15—C16	−0.4 (4)	C6—C7—C8—C18	179.48 (18)
N11—N12—N13—C14	1.1 (2)	C7—C8—C8A—N1	−175.58 (18)
N12—N13—C14—C1	176.0 (2)	C7—C8—C8A—C4A	4.1 (3)
N12—N13—C14—C15	0.4 (3)	C18—C8—C8A—N1	4.3 (3)
C23A—N24A—C1—O1	−2.2 (5)	C18—C8—C8A—C4A	−176.01 (17)
C23A—N24A—C1—C14	−167.5 (3)	C7—C8—C18—F1	−5.8 (3)
C25A—N24A—C1—O1	173.2 (4)	C7—C8—C18—F2	114.3 (2)
C25A—N24A—C1—C14	7.9 (6)	C7—C8—C18—F3	−125.7 (2)
C1—N24A—C23A—C22A	−134.2 (6)	C8A—C8—C18—F1	174.31 (17)
C25A—N24A—C23A—C22A	49.7 (7)	C8A—C8—C18—F2	−65.7 (3)
C1—N24A—C25A—C26A	135.5 (6)	C8A—C8—C18—F3	54.4 (3)
C23A—N24A—C25A—C26A	−49.0 (7)	N13—C14—C15—N11	−1.7 (2)
O1—C1—C14—N13	−150.7 (2)	N13—C14—C15—C16	174.5 (2)
O1—C1—C14—C15	24.3 (4)	C1—C14—C15—N11	−177.3 (2)
N24A—C1—C14—N13	15.3 (4)	C1—C14—C15—C16	−1.2 (4)
N24A—C1—C14—C15	−169.7 (3)	O21—C22A—C23A—N24A	−52.0 (9)
N1—C2—C3—C4	−0.7 (4)	N24A—C25A—C26A—O21	49.3 (9)
C2—C3—C4—N11	177.1 (2)		

Symmetry codes: (i) *x*−1, *y*, *z*; (ii) −*x*, −*y*+1, −*z*; (iii) −*x*−1, −*y*, −*z*; (iv) *x*−1, *y*−1, *z*−1; (v) *x*−1, *y*−1, *z*; (vi) −*x*, −*y*, −*z*; (vii) −*x*+1, −*y*+1, −*z*+1; (viii) −*x*+1, −*y*+2, −*z*+1; (ix) −*x*+2, −*y*+2, −*z*+1; (x) *x*+1, *y*, *z*; (xi) −*x*, −*y*+1, −*z*+1; (xii) *x*+1, *y*+1, *z*+1; (xiii) *x*+1, *y*+1, *z*.

### Hydrogen-bond geometry (Å, °)

**Table:**

*D*—H···*A*	*D*—H	H···*A*	*D*···*A*	*D*—H···*A*
C3—H3···F1^x^	0.95	2.35	3.237 (3)	156
C7—H7···F1	0.95	2.37	2.703 (3)	100
C16—H16B···O1	0.98	2.44	3.012 (3)	117
C23A—H23B···O1	0.99	2.36	2.812 (5)	107
C25A—H25A···N13	0.99	2.25	2.893 (5)	122

Symmetry codes: (x) *x*+1, *y*, *z*.
